# Quality assurance via telephone interviews after cataract surgery: An explorative study

**DOI:** 10.1371/journal.pone.0298149

**Published:** 2024-03-07

**Authors:** Manuel Ruiss, Viktoria Pai, Caroline Pilwachs, Natascha Bayer, Stefan Palkovits, Oliver Findl

**Affiliations:** Vienna Institute for Research in Ocular Surgery (VIROS), a Karl Landsteiner Institute, Department of Ophthalmology, Hanusch Hospital, Vienna, Austria; University of Warmia, POLAND

## Abstract

**Introduction:**

Cataract surgery is a relatively safe procedure with satisfactory postoperative results in most patients. However, in rare cases severe complications can occur shortly after the intervention. Therefore, patients are advised to undergo an ophthalmological examination postoperatively, which should be performed as soon as possible in case of emergencies. However, exactly when these follow-up visits should take place is still discussed. A time- and cost-saving alternative to this could be short-term postoperative telemedical approaches. The aim of this study was to analyze patient complaints as well as satisfaction with and the best timepoint to perform telephone calls after cataract surgery.

**Methods:**

Patients scheduled for cataract surgery received a telephone call on the surgery day or the day after (study group) during which they were asked about complaints or additional examination visits. Patients without telephone calls served as control group. All patients had a follow-up visit one week after the intervention during which a questionnaire was filled out and the study group was asked about their satisfaction with the telephone calls.

**Results:**

181 patients were recruited in this study. Ocular surface problems were the most common postoperative symptom. More than 80% of the patients were very satisfied with the telephone calls, with patients being contacted on the day of surgery being more calmed than those called on the next day. No difference in additional and planned follow-up visits was found between the study and the control group (P > .40). Postoperative patient complaints (Phi 0.372, P < .001) and additional prescribed therapy (Phi 0.480, P < .001) were moderately associated with additional visits.

**Conclusion:**

Satisfaction with telephone reviews shortly after cataract surgery was very high and contacting patients on the evening of the day of the procedure could be a time- and cost-saving alternative to short-term in-house follow-up visits.

## Introduction

Cataract surgery is one of the most frequently performed elective surgeries worldwide [1]. It is a fast and relatively safe procedure that yields good postoperative visual results in most cases. The European Registry of Quality Outcomes for Cataract and Refractive Surgery (EUREQUO) reported that in more than 2,7 million cataract surgery patients a reduction in the surgical complication rates from 2.5% to 1.2% was seen between 2008 and 2017 [2]. Therefore, due to the recent improvements in the techniques and materials used in the procedure, most patients can leave the hospital the same day after an uncomplicated intervention.

In the EUREQUO it was further shown that 1.88% of 167,366 patients in 2017 had short-time postoperative complications during a 30-day follow-up (FU) period [2]. Although rarely seen, serious complications including cystoid macular edema, retinal detachment, or endophthalmitis might occur shortly after the procedure [3–5]. Therefore, it is recommended that all patients should have an ophthalmological examination during the 1-week postoperative period or as soon as possible in case of emergencies. However, there is an ongoing debate as to whether this FU visit is necessary and when it should best take place. One recommendation, according to the Cataract in the Adult Eye Preferred Practice Pattern® guidelines published by the American Academy of Ophthalmology (AAO), is that a postsurgical control within 24 hours should be done for patients with preoperative risk factors [e.g., high intraocular pressure (IOP)] or any surgical complications, whereas for those without problems or complaints 48 hours are sufficient. Additionally, in the former cases more frequent FUs are recommended, while in the latter cases without problems the frequency of the visits depends on different factors like visual outcome or any other existing eye problems. However, in case of symptoms that indicate possible endophthalmitis like pain, reduction in vision, redness, etc., a prompt visit at an ophthalmologist is necessary [6].

During the patient-doctor interview at the pre-examination for cataract surgery, all patients receive information about possible postsurgical emergencies. However, some patients may still be uncertain whether it is necessary to see a doctor in case complaints occur after the intervention. Additionally, during the COVID-19 pandemic access to ophthalmic services was compromised and since most cataract surgery patients belong to the vulnerable group, ophthalmological FU visits might have been avoided [7]. Further reasons for missing after-care visits are the patient’s age and a longer distance from the hospital [8].

Telemedicine approaches instead of in-house FUs might be a time- and cost-saving way to overcome these problems. Therefore, this study aimed to analyse the patient’s satisfaction with telephone calls to monitor patients’ complaints shortly after cataract surgery and to find out the appropriate time point to perform them.

## Materials and methods

This randomized controlled single-center trial included patients older than 18 years scheduled for bilateral cataract surgery. People were excluded from the study if they were not literate in the German language or did not have a telephone. All research and measures of this study complied with the tenets of the Declaration of Helsinki and were approved by the ethics committee of the city of Vienna (approval number: EK 19-192-VK). After informing each participant about the nature of the study, written informed consent was signed before enrolment into the trial and before any study-specific measures were carried out. The study was registered at clinicaltrials.gov (https://clinicaltrials.gov) with the identification number NCT05215002.

Patients were recruited at the department of Ophthalmology of the Hanusch hospital, Vienna, during a period between 02/12/2019 and 10/05/2022. The study was temporarily interrupted starting with the first COVID-19 lockdown in 03/2020 and re-started in 01/2022. In all patients, a face-to-face interview with an ophthalmologist and a routine ophthalmological examination was performed 1 week before cataract surgery of the first eye. During this visit all patients were informed by a physician about intra- and postoperative complications and an educational sheet including a 24-hour emergency telephone number was handed out to them. After signing the informed consent for participating in the study, the patients were randomized into either a study (“telephone”) group or a control group in a 1:1 fashion by using an online randomization tool (http://www.randomizer.org; accessed December 2019). Study participants were not told which group they were assigned to. In all patients, routine phacoemulsification surgery in local anesthesia with implantation of either a monofocal or a monofocal toric intraocular lens (IOL) was performed. The time between the surgery of the first eye and the second eye was 1 week. The patients received a transparent plastic shield to be worn during the day of surgery and the first night. Bromfenac eye drops (Bausch & Lomb, USA) twice a day for 4 weeks was prescribed for every patient. Preoperative risk factors, intraoperative complications, and immediate additional therapy directly after surgery were assessed for each patient.

### Telephone calls

Patients allocated to study group A were called on the late afternoon and evening of the day of surgery, while patients in study group B were called in the morning of the day after the surgery. A total of 3 attempts to reach a patient per telephone were made, after which the patent was excluded from the study. All telephone calls were performed by the same trained person of our medical staff.

During the telephone calls, all participants were asked about any postoperative symptoms. In case the patients stated any complaints, they were asked in a more detailed way and all symptoms were noted on a case report form. Complaints such as itching, burning, or tearing of the eye were summarized as ocular surface problems. Care was taken that the questions were formulated in an easily understandable fashion.

The participants were then asked if they had already scheduled an appointment at an ophthalmologist’s office or a clinic within the first week after the surgery (“planned” visit). Further, if they had any additional visit due to acute complaints (“additional” visit). Reasons for additional visits and the place where they were done were noted. In case patients mentioned any serious complaints (e.g., severe pain, vision loss) during the phone calls, an immediate FU visit at our department was recommended (“recommended” visit).

### Follow-up visit one week after the surgery

**Fig 1:**
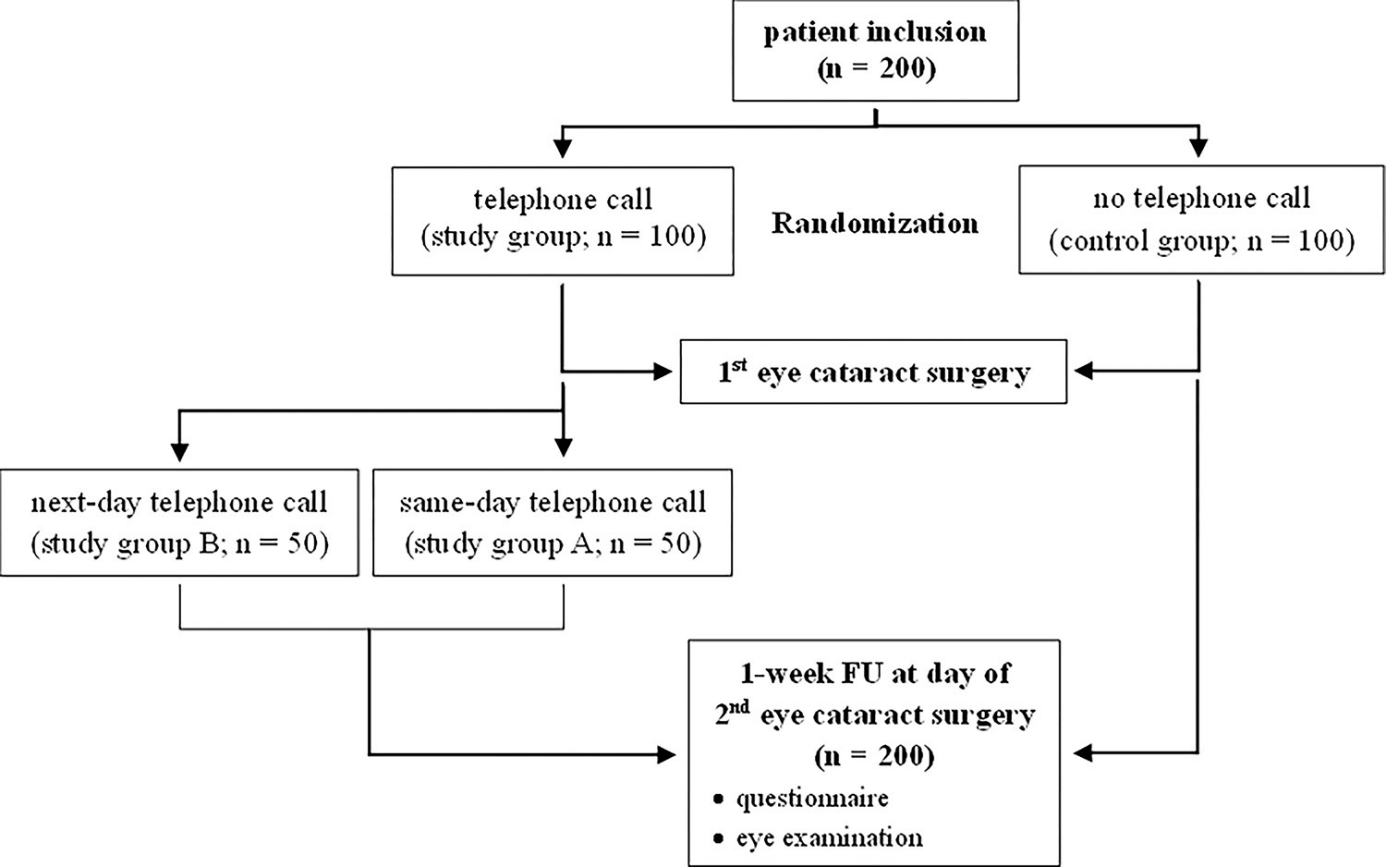
Planned workflow for the study and the control group.

One week after first eye cataract surgery participants of the study and control group were asked to answer a questionnaire concerning their postoperative complaints, additional visits within the week, and any prescribed medications. This was done before any other pre-surgical eye examinations or doctor-patient interviews took place (Fig 1). If the patients had any questions concerning the questionnaire, one person from the medical staff involved in the study was available. Patients of the study group were asked to answer an additional set of questions concerning their satisfaction with the phone calls, if they would prefer being called after any future other ophthalmological surgery, and if the call calmed or unsettled them. Further, participants were offered the possibility to add any additional information if they wanted.

### Statistical analyses

All statistical analyses were performed using Excel 2016 (Microsoft Corporation, USA) and SPSS Statistics version 26.0 (IBM Corporation, USA). Descriptive data are shown either as median, mean, and range or in the case of categorical variables as number and percentage. To test for normal distribution of the outcome parameters, the Shapiro-Wilk test and graphical analysis via histograms were performed. Fisher’s exact test was used to detect differences between categorical data and correlation analysis between the different categorial parameters was done using the Phi coefficient. A P value < .05 was considered statistically significant.

## Results

200 patients were recruited into this study, of which 19 patients were lost to follow-up (17 could not be reached by telephone and in 2 cases the surgery was cancelled). Therefore, a total of 181 participants, 89 in the study group and 92 in the control group, were included in the analysis. Of the 89 patients in the study group, 46 were allocated to study group A (time between surgery and call: 6:22 hours, range: 4:53 to 7:38 hours) and the other 43 to study group B (time between surgery and call: 24 ± 2 hours). 58 (65%) participants in the study group and 48 (52%) of the participants in the control group were female. The median age was 72 years (range: 49 to 91 years) and 73 years (range: 47 to 96 years), respectively. In 13 (15%) patients of the study group and 10 (11%) patients of the control group a monofocal toric IOL was implanted (Table 1). No difference in preoperative surgical risk factors, intraoperative complications, and immediate postoperative additional therapy was found between the study and the control group (all P > .20, Table 2).

**Table 1:** Baseline demographics of the study participants in the control and the study group.

Parameter	study group (n = 89)	control group (n = 92)
patient number	100	100
excluded	11	8
age (years)	72.0 (72.0, 49.0 to 91.0)	73.0 (72.3, 47.0 to 96.0)
female: male	58 (65): 31 (35)	48 (52): 44 (48)
eye to be operated		
od: os	51 (57): 38 (43)	56 (61): 36 (39)
IOL type		
monofocal: toric	76 (85): 13 (15)	82 (89): 10 (11)

Values are shown as median (mean, range) or number (%). IOL = intraocular lens; n = number of patients; od = oculus dexter; os = oculus sinister.

**Table 2:** Risk factors, intraoperative complications, and immediate postoperative additional therapy of the study participants.

Parameter	study group (n = 89)	control group (n = 92)	P value
**preoperative surgical risk factors**	30 (34)	27 (29)	.678
> 1 risk factor	6 (7)	3 (3)	.325
mature cataract	10 (11)	6 (7)	.303
shallow anterior chamber	7 (8)	9 (10)	.795
α-antagonist intake/ small pupil	6 (7)	3 (3)	.325
PEX	7 (8)	6 (7)	.780
cornea guttata/ opacification	6 (7)	6 (7)	1.000
posterior synechiae	1 (1)	1 (1)	1.000
status post laser vision correction	0	1 (1)	1.000
**intraoperative complications**	6 (7)	13 (14)	.429
IFIS	4 (4)	9 (10)	.250
corneal erosion	0	2 (2)	.497
corneal opacification	1 (1)	0	.492
keratitis punctata superficialis	1 (1)	0	.492
capsular rupture	0	2 (2)	.497
**immediate postoperative additional therapy**	35 (39)	31 (34)	.678
> 1 therapy	4 (4)	5 (5)	1.000
lubricants	5 (6)	5 (5)	1.000
anti-inflammatory	22 (25)	18 (20)	.375
anti-glaucoma	12 (13)	11 (12)	.825
analgesic	0	1 (1)	1.000
bandage contact lens	0	1 (1)	1.000

Values are shown as numbers (%). Percentage is given as percent of total patient numbers per group. IFIS = intraoperative floppy iris syndrome; n = number of patients; PEX = pseudoexfoliation syndrome. Fisher’s exact test was used to test for statistically significant differences. A P value < .05 was considered statistically significant.

Postsurgical symptoms were reported by 24 (52%) patients in study group A and 18 (42%) in study group B (P = .397), with surface problems being the most prevalent. A lower number of symptoms was reported by the same patients when they were asked again 1 week after the surgery [11 (24%) in study group A, 14 (33%) in study group B], with a statistically significant difference only for study group A (P = .009). Again, surface problems were stated as the most prevalent complaints in both study cohorts. When looking at both study subgroups together, a change in surface problems from 36% immediately after surgery to 17% after 1 week was seen (P < .001). Although not significant, fewer complaints, except for decrease in visual acuity and positive dysphotopsia, were reported in study group A compared to study group B. No significant difference was found in reported postoperative symptoms between the study and the control group [25 (28%) vs. 22 (24%), P = .612] (Table 3).

**Table 3:** Patients’ complaints reported during the telephone calls and 1 week postoperatively.

Parameter	postoperative telephone calls			1 week postoperative			
	same day call (n = 46)	next day call (n = 43)	P value	same day call (n = 46)	next day call (n = 43)	control group (n = 92)	P value^‡^
complaints	24 (52)	18 (42)	.397	11 (24)	14 (33)	22 (24)	.612
> 1 complaint	4 (9)	5 (12)	.743	3 (7)	2 (5)	6 (7)	1.000
surface problems	20 (43)	12 (28)	.184	7 (15)	8 (19)	14 (15)	.841
eye pain	5 (11)	3 (7)	.715	2 (4)	3 (7)	5 (5)	1.000
eye redness	2 (4)	1 (2)	1.000	1 (2)	0	2 (2)	1.000
dark shadows/ dots	0	0	N/A	1 (2)	1 (2)	1 (1)	.617
decreased visual acuity	0	4 (9)	.051	1 (2)	0	3 (3)	.621
light arc	0	3 (7)	.109	1 (2)	0	1 (1)	1.000
headache	1 (2)	0	1.000	0	0	1 (1)	1.000
flicker/ flashing lights	0	0	N/A	1 (2)	0	0	.492
other	1 (2)	3 (7)	.350	0	4 (9)	1 (1)	.206

Values are shown as numbers (%). Percentage is given as percent of total patient numbers per group. N/A = not applicable; n = number. Fisher’s exact test was used to test for statistically significant differences. ^‡^ P values for the 1-week postoperative visit are shown between the control group and the pooled study group (same day call and next day call together). A P value < .05 was considered statistically significant.

**Fig 2:**
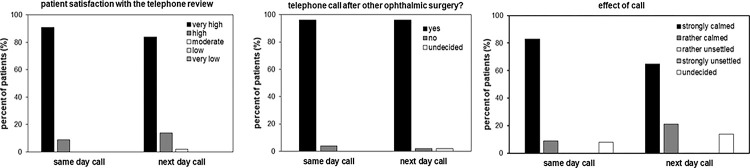
Patient attitudes concerning the postsurgical telephone calls.

Very high satisfaction with the telephone calls was stated by 42 (91%) patients in study group A and 36 (84%) of those in study group B. Of both groups, more than 95% would like to have such a call in the future for any other ophthalmic surgery. Also, 38 (83%) patients in study group A and 28 (65%) in study group B were strongly calmed by the telephone call, whereas no participant in any group stated to be rather or strongly unsettled by the call (Fig 2).

About half of the patients of both, the study and the control, groups did not attend any planned or additional visit during the first week after the cataract surgery (P = .373). A planned follow-up visit was stated by 39% (n = 35) of the study group and 43% (n = 40) of the control group, of which most were done at their referring ophthalmologist’s office (all P > .40). In 20 (11%) participants planned visits were scheduled because of a postoperative intraocular pressure (IOP) control. An additional visit was necessary for 6 (7%) patients in the study group and 7 (8%) in the control group. Reasons therefore were corneal erosion, red eye, eye pain, visual acuity decrease, seeing black dots, eye tearing, and negative dysphotopsia. A visit was recommended to three patients in study group B. No significant differences were seen in visits as well as additional therapy between the study and control groups within the first postoperative week (all P > .10) [Table 4].

**Table 4:** Planned, unplanned, and recommended postoperative follow-up visits and additional prescribed therapy during the controls.

Parameter	postoperative telephone calls			1 week postoperative			
	same day call (n = 46)	next day call (n = 43)	P value	same day call (n = 46)	next day call (n = 43)	control group (n = 92)	P value^‡^
> 1 visit	5 (11)	1 (2)	.204	2 (4)	1 (2)	1 (1)	.363
no control during 1 week	30 (65)	25 (58)	.520	24 (52)	27 (63)	46 (50)	.373
planned control	21 (46)	18 (42)	.519	21 (46)	14 (33)	40 (43)	.449
clinic	2 (4)	2 (5)	1.000	6 (13)	4 (9)	10 (11)	1.000
ophthalmologist	19 (41)	16 (37)	.666	15 (33)	10 (23)	30 (33)	.752
unplanned control	0	1 (2)	.483	3 (7)	3 (7)	7 (8)	1.000
clinic	0	0	N/A	1 (2)	0	5 (5)	.211
ophthalmologist	0	1 (2)	.483	2 (4)	3 (7)	2 (2)	.383
recommended control	0	3 (7)	.109	0	1 (2)	N/A	N/A
additional therapy during controls	N/A	N/A	N/A	5 (11)	0	9 (10)	.561

Values are shown as numbers (%). Percentage is given as percent of total patient numbers per group. N/A = not applicable; n = number. Fisher’s exact test was used to test for statistically significant differences.

^‡^ P values for the 1-week postoperative visit are shown between the control group and the pooled study group (same day call and next day call together). A P value < .05 was considered statistically significant.

No significant correlation was found between preoperative risk factors, intraoperative complications, and immediate postoperative additional therapy when compared with postoperative patient complaints, additional visits, or recommended visits (all Phi < 0.10, P > .30). Patients with postoperative complaints (Phi 0.372, P < .001) and those receiving additional therapy (Phi 0.480, P < .001) were more likely to attend an additional visit, but there was only a non-significant association of both parameters with planned or recommended visits (all Phi < 0.1, P > .30).

## Discussion

Telephone reviews shortly after cataract surgery yield very high patient satisfaction and may be even more calming if conducted in the afternoon or evening after the intervention.

No difference in postoperative symptoms were seen between the groups and no serious adverse events (SAEs) were detected in this patient cohort. The most common short-term complaints after cataract surgery were ocular surface problems, which were significantly less one week after surgery. These results are comparable to those of Elksnis et al. [9], who suggested that dry eye symptoms rather occur during the early postoperative period, whereas Iglesias et al. [10] reported that 32% of their patients had dry eye symptoms even 6 months after the procedure. These findings are reflected by our data, as all of these results show that ocular surface problems seem to concern many patients and should be treated to improve their postoperative experience.

In our cohort, the patient satisfaction with telephone calls shortly after cataract surgery was very high. However, more participants in the group contacted on the day of surgery stated that the method calmed them very much compared to those that were called on the morning of the fist postoperative day. Interestingly, the same-day call cohort reported more postoperative complaints during the telephone calls. This might be due to issues of the ocular surface as well as irritation or due to the surgical process itself, which may alleviate during the first night after surgery. Additionally, due to the psychological stress on the day of surgery, patients possibly pay more attention to any occurring symptoms. Both assumptions are further supported by the finding that patients in the same-day call group reported significantly fewer postoperative symptoms when asked one week after the procedure than stated during the phone calls. According to this information, a phone call immediately on the day of the surgery could be more useful to calm the patients and to avoid any unnecessary visits to the hospital compared to contacting the patients the day after.

A comparable number of additional postoperative FUs were attended by patients of both groups. A reason may be a too small number of study participants to detect differences since cataract surgery today is a relatively safe procedure and SAEs occur rarely (e.g., 0.01% endophthalmitis cases in 167,366 cataract surgery patients in 2017 [2]). Furthermore, in our clinic, all cataract patients get informed about possible postoperative emergencies and how to behave in such a situation by written information and during the patient-doctor informed consent discussion. Additionally, about half of all patients had already made a postoperative appointment with their ophthalmologists and, hence, might have hesitated to attend an unscheduled visit in case of complaints. On the other hand, the lack of difference between additional visits between the groups, is an indication that the telephone calls did not scare or confuse the patients.

During the telephone FUs, three patients were advised to see an ophthalmologist, of which only one patient who noticed flashes of bright light attended an examination at our department. Although only positive dysphotopsia were diagnosed, flashes can be a symptom of more serious problems like retinal detachment. This example shows that telephone calls might be an important decision help for patients since this participant was very anxious about the symptom, stated that he was unsure of what to do, and that he only visited our department because of the call.

Moustafa et al. [11] reported that positive answers in a standardized set of questions (e.g., pain, redness, decrease in vision, etc.) 1 week after cataract surgery were associated with a higher incidence of unexpected postoperative management changes. It was suggested that these questions might be used as a predictor for patients needing postoperative follow-ups and that they could also be delivered via telephone. Interestingly, we did not find an association between postoperative symptoms and additional therapy prescribed during the 1-week period after surgery in our patient cohort. Only additional visits were moderately correlated with postoperative management changes. However, contrary to the study by Moustafa et al., we also analyzed patients with preoperative risk factors and intraoperative complications, while in their study a low-risk cohort with uncomplicated surgeries was included. Zemaitiene et al. [12], who used the same set of questions as Moustafa et al. in a low-risk patient cohort with uncomplicated surgeries, also did not find an association between positive answers and unexpected management changes. Furthermore, additional visits, recommended visits, and postoperative complaints were not associated with preoperative risk factors, intraoperative complications, or additional therapy immediately subscribed after surgery in our patient cohort.

Telephone calls to monitor patient safety have already been described for different other surgeries such as lid and lacrimal surgery [13], colorectal surgery [14], orthopaedic surgery [15], vascular surgery [16], congenital heart surgery [17], caesarean section [18], and pelvic surgery [19]. Additionally, telephone reviews were also evaluated after phacoemulsification surgery in different clinical trials. Mandal et al. [20] found that telephone calls 1 day after cataract surgery were a good alternative to home and in-hospital FU visits. Tan et al. [21] reported that a telephone questionnaire one day after cataract surgery was a safe and effective postoperative review method and comparable results were shown by Hoffman & Pelosini [22] even after a longer period (12–24 days). However, contrary to our study, in all of these trials only low-risk patients with uneventful cataract surgery were included and the trials were either non-randomized, had no control groups, lower patient numbers, no same-day calls, or included predominantly second eye surgery patients.

The advantages of telephone calls as a telemedical approach for postsurgical FUs are the low requirements for the patient and the interviewer, increased patient satisfaction (e.g., no hospital travel, no waiting time, more time to ask questions, decisional help), and reduction in costs as well as unplanned postoperative visits for the hospital. Additionally, telephone monitoring could also be combined with other telemedical approaches like smartphone applications or digital visual acuity testing tools. A further development, which is currently under research, is the use of an artificial intelligence (AI)-enabled autonomous telemedicine call system, which is supposed to make an automatic call between 25 and 27 days after cataract surgery supervised by an ophthalmology research fellow [23]. Disadvantages of this approach are that SAEs might be overseen, that referring ophthalmologists do not see their patients after surgery, and a loss in learning opportunities for trainees. Also, telephone calls might exclude patients with a hearing deficit, language barriers, cognitive disorders, or poor phone reception. However, we did not experience such problems in any of our 89 study patients.

In summary, our study showed high satisfaction with telephone consultation for patient monitoring shortly after cataract surgery. It might be more useful to perform the calls a few hours after the procedure than on the next day. Postoperative telephone reviews appear to be a good approach to decide which patients should be examined by an ophthalmologist and which patients do not need any further check-up in the early postoperative phase.
